# Jumping the green wall: The use of PNA‐DNA clamps to enhance microbiome sampling depth in wildlife microbiome research

**DOI:** 10.1002/ece3.6814

**Published:** 2020-09-24

**Authors:** Luis Víquez‐R, Ramona Fleischer, Kerstin Wilhelm, Marco Tschapka, Simone Sommer

**Affiliations:** ^1^ Institute for Evolutionary Ecology and Conservation Genomics University of Ulm Ulm Germany; ^2^ Smithsonian Tropical Research Institute Balboa Panama

**Keywords:** 16s, chloroplast, mitochondria, PCR blockers, PNAs, wildlife microbiome

## Abstract

As microbiome research moves away from model organisms to wildlife, new challenges for microbiome high‐throughput sequencing arise caused by the variety of wildlife diets. High levels of contamination are commonly observed emanating from the host (mitochondria) or diet (chloroplast). Such high contamination levels affect the overall sequencing depth of wildlife samples thus decreasing statistical power and leading to poor performance in downstream analysis. We developed an amplification protocol utilizing PNA‐DNA clamps to maximize the use of resources and to increase the sampling depth of true microbiome sequences in samples with high levels of plastid contamination. We chose two study organisms, a bat (*Leptonyteris yerbabuenae*) and a bird (*Mimus parvulus*), both relying on heavy plant‐based diets that sometimes lead to traces of plant‐based fecal material producing high contamination signals from chloroplasts and mitochondria. On average, our protocol yielded a 13‐fold increase in bacterial sequence amplification compared with the standard protocol (Earth Microbiome Protocol) used in wildlife research. For both focal species, we were able to increase significantly the percentage of sequences available for downstream analyses after the filtering of plastids and mitochondria. Our study presents the first results obtained by using PNA‐DNA clamps to block the PCR amplification of chloroplast and mitochondrial DNA from the diet in the gut microbiome of wildlife. The method involves a cost‐effective molecular technique instead of the filtering out of unwanted sequencing reads. As 33% and 26% of birds and bats, respectively, have a plant‐based diet, the tool that we present here will optimize the sequencing and analysis of wild microbiomes.

## Graphical abstract

We develop a new tool to prevent contaminating mitochondrial and chloroplast DNA in wildlife gut microbiome sequencing, yielding on average a 13‐fold increase in usable reads. Our method allows for a more effective use of sequencing resources in wildlife microbiome research.

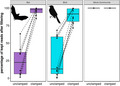

## 1 INTRODUCTION

A new world of research opportunities has emerged with the advancement of sequencing techniques. One of the fields that have benefited most is the study of whole microbial communities, so‐called microbiomes. This method allows the study of microbial communities, including those closely associated with eukaryotic hosts, without the need to cultivate each bacterium separately (Caporaso et al., 2012). Together with recently developed and improved bioinformatic pipelines (Mothur, QIIME 2, etc.), we now have the means to classify and assign taxonomy with a reasonable level of confidence (Bolyen et al., 2019).

As microbiome research moves away from model organisms and extends into natural settings, new challenges of wildlife research and those arising because of the variety of wildlife diets need to be tackled. One of the challenges is the separation of bacterial from nonbacterial sequences, that is, those from mitochondria (from the host) and chloroplasts (from the diet) can sometimes be tricky (Barott et al., 2011; Lundberg et al., 2013). According to the widely accepted endosymbiosis theory (Margulis [then known as Sagan (1967), Gray 2017], mitochondria and chloroplasts were originally derived during evolution from hijacked bacteria engulfed by other bacteria. Because of this bacterial origin, some DNA sequences of organelles are strikingly bacteria‐like. This is also the case with reads obtained from high‐throughput sequencing of 16S rRNA genes, the usual target of microbiome studies. In the worst case, the resulting read coverage consists of many reads assigned to mitochondria or chloroplasts.

Several ways are available to circumvent this problem; the most common path is to increase the sequencing depth and then filter out the reads assigned to the organelles. However, this technique results in an expensive price tag (due to the high percentage of reads wasted on contamination) for sequencing and may lead to highly skewed read numbers depending on the provenance of the samples. Another option has recently arisen: the use of DNA‐PNA clamps as PCR blockers to prevent the amplification of the specific mitochondrial or chloroplast sequences (Lundberg et al., 2013). PNAs (peptide nucleic acids) are DNA‐mimicking molecules with outstanding hybridization properties (Nielsen & Egholm, 1999). The backbone of the molecules is constructed of N‐(2‐amino‐ethyl) glycyl (AEG) instead of the sugar‐phosphate backbone of DNA (Nielsen et al., 1994). The nucleobases attached to this backbone are the same as those in DNA, thereby allowing hybridization between the probe and the bacterial DNA. PNAs are thus a powerful molecular tool in microbiome research for dealing with samples with a high content of either host or plant remnants in fecal pellets (Fitzpatrick et al., 2018; Lundberg et al., 2013).

In this study, we tested the PNA‐DNA clamps as a method for improving microbiome discovery rates in bats (tequila bat *Leptonycteris yerbabuenae*) and Galapagos mockingbirds (*Mimus parvulus*). We chose these two study organisms because they both rely on heavy plant‐based diets that sometimes can lead to masses of plant‐based fecal material producing high contamination signals from chloroplast and mitochondria. Our study presents the first results obtained by using PNA‐DNA clamps to block the PCR amplification of chloroplast and mitochondrial DNA from the diet during investigations of gut microbiomes of wild animal populations. The method involves a cost‐effective molecular technique, instead of the filtering out of the unwanted sequencing reads.

## 2 MATERIALS AND METHODS

### 2.1 Sample collection

In 2018, we netted tequila bats (*L. yerbabuenae*) while they were returning from a night's foraging trip. A mist net was positioned at the entrance of the roosting cave located in the Pinacate and Gran Desierto de Altar Biosphere Reserve in Northern Sonora, Mexico. Bats were immediately removed from the net and kept in a soft cloth bag until processed (<60 min). Animals were handled following guidelines from the ASM for animal care (Sikes & The animal care & use committee of the American society of Mammalogists, 2016) and local regulations (Permit Number: SGPA/DGVS/06361/17). A single fecal pellet was collected from the cloth bag and preserved in a safe‐lock 1.5‐ml Eppendorf tube containing 500 µl DNA/RNA shield (Zymo Research Europe GmbH, Germany). The tube was shaken to ensure the maximum impregnation of the sample with the buffer and then stored in a cool place until it could be frozen at −20°C. For the present study, we used samples from eight randomly chosen individuals.

Mockingbird (*M. parvulus*) individuals were captured between 2007 and 2008 at various sites across the Galapagos Islands. Birds were trapped by using mist nets or potter traps. Fecal pellets from the birds were collected in ethanol and stored at −20°C. Further details about the capturing procedure are given in Hoeck et al. (2010) and Štefka et al. (2011). In the present study, we used samples from ten randomly chosen individuals inhabiting the islands of Santiago, Santa Cruz, and Marchena (Fleischer et al., in review).

### 2.2 DNA extraction

We extracted the fecal pellets by using the NucleoSpin^®^ Soil extraction kit (Macherey‐Nagel, Düren, Germany) following the manufacturer's guidelines. For the tequila bat samples, we homogenized the sample (2 × 150 s at 50 Hz) by using a SpeedMill PLUS (Analytik Jena, Jena, Germany). To maximize DNA yield, we conducted consecutive elutions (2 × 50 µl) with a preheated (ca. 45°C) SE buffer. For the mockingbird samples, the samples were washed in 50 µl SE buffer and then homogenized using the same procedure as with the tequila bat samples. We stored the extracted DNA at −20°C.

### 2.3 PNA‐DNA clamp design

The probes in our study were designed based on the work of Lundberg et al. (2013) who developed PNA‐DNA clamps to block mitochondrial (mPNA) and chloroplast (pPNA) DNA, these clamps are known as universal clamps. Recently, Fitzpatrick et al. (2018) reported that the universal pPNA showed a mismatch in six plant lineages by means of an experimental and bioinformatic analysis. Preliminary results from our study showed that the plant contamination material in our bat samples belonged to one of these lineages, namely Saguaro Columnar cacti (Cactaceae: *Carnigea gigantea*). Therefore, following the recommendations of Fitzpatrick et al. (2018), we developed a special clamp for the bat samples (cpPNA: 5′GGCTCAACCCCGGACAG‐3′); the sequence for the universal PNA‐DNA clamps (cPNA and mPNA) can be obtained from Lundberg et al. (2013). This is not a trivial matter, since even a single base mismatch between the chloroplast sequence and the clamp can increase levels of plastid contamination in the sequencing output (Fitzpatrick et al., 2018). For the mockingbird, the universal clamps were used to block both chloroplast and mitochondrial DNA. All clamps were ordered from PNA Bio (Newbury Park, USA).

### 2.4 DNA amplification, library preparation, and sequencing

To investigate the gut microbiomes of bats (*n* = 8) and birds (*n* = 10), we followed the Earth Microbiome Protocol (Caporaso et al., 2010). Moreover, we added four samples consisting of a ZymoBIOMICS microbial community standard D6300 (Zymo Research Europe, Freiburg, Germany). These were used as positive controls for microbiome amplification and allowed us to examine whether the clamps had any effect over the yield of a normal sample depleted of chloroplast and mitochondria. The extracted DNA was amplified with the universal bacterial primers 515F (5′‐GTGCCAGCMGCCGCGGTAA‐3′) and 806R (5′‐GGACTACHVGGGTWTCTAAT‐3′). We used a two‐step amplification process following the amplicon tagging scheme of Fluidigm (Access Array System™ for Illumina Sequencing Systems, ©Fluidigm, San Francisco, USA). In the first step, we amplified a 291‐bp fragment of the hypervariable V4 region of the 16S rRNA gene by using tagged (CS) target‐specific (TS) primers: CS1‐NNNN‐TS‐515F and CS2‐TS‐806R. We added four random bases to our forward primers to facilitate cluster identification during the first cycles on the Illumina MiSeq System. In the second step, the tags (CS1 and CS2) were used to add a sample‐specific 10 bp barcode and the Illumina system adapters.

**Figure 1:**
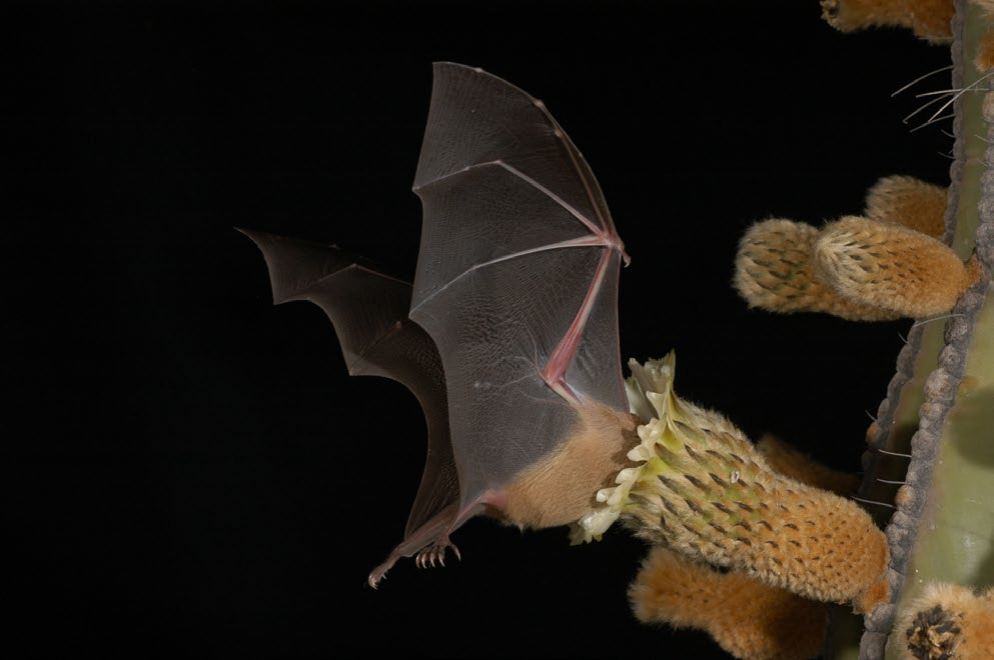
A tequila bat (*Leptonycteris yerbabuenae*) visiting a cactus flower (image for journal cover)

The initial 15 μl PCR volume contained 1.5 μl (5–15 ng) extracted DNA, 7.5 μl DNA polymerase AmpliTaq Gold™ 360 Master Mix (Applied Biosystems, Darmstadt, Germany), 1.5 μl (0.2 μM) primers, and 4.5 μl sterile water. The PCR protocol consisted of an initial activation step at 95°C for 10 min, followed by 35 cycles at 95°C for 30 s, 60°C for 30 s and 72°C for 45 s, and a final elongation at 72°C for 10 min. When clamps where implemented, the water volume was reduced to 1.5 μl; the 1.5 μl from each clamp (mPNA and either cpPNA or pPNA) was added to this first step to give a final concentration of 1 μM (Figure 1).

**Figure 2:**
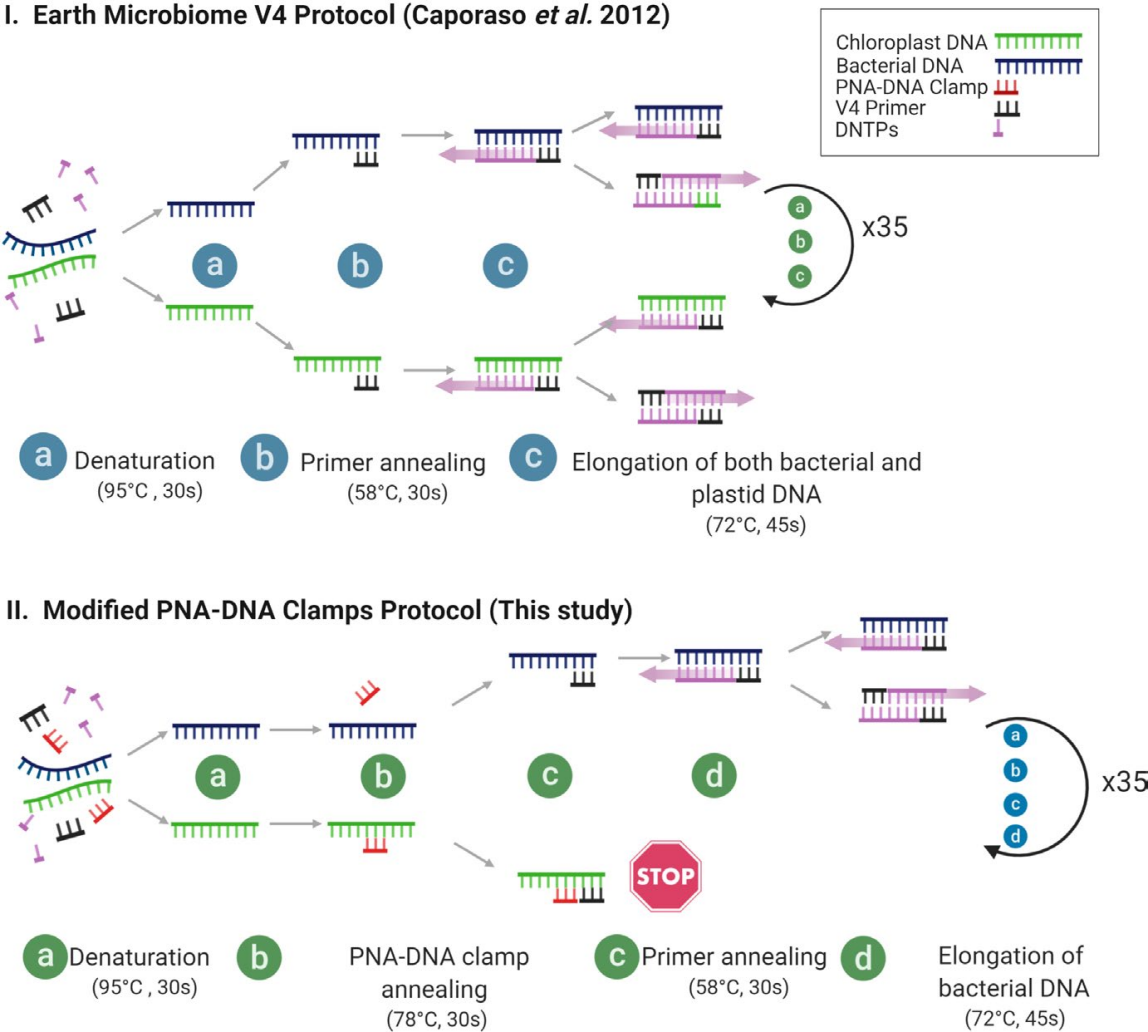
PCR protocol for the implementations of the cpPNA‐DNA clamps. I. Normal workflow according to the Earth Microbiome Protocol (Caporaso et al., 2012); and II. our modified protocol with the addition of one extra step for clamp annealing (Step b)

The modified PCR protocol included a step in order to allow the binding of the PNA to the target sequences (Figure 2). For the second barcoding PCR (20 μl), we used 3 μl initial PCR product, 10 μl AmpliTaq Gold™ 360 Master Mix, 4 μl (0.4 μM) barcode primers (Fluidigm), and 3 μl sterile water. PCR conditions were the same as before, but only 10 cycles were performed.

We used the NucleoMag^®^ NGS Clean‐Up and Size Select Kit (Macherey‐Nagel, Düren, Germany) on a GeneTheatre^®^ (Analytik Jena, Jena, Germany) to clean the PCR products according to the manufacturer's guidelines. We assessed the quality of the amplicons by using the QIAxcel Advanced System^®^ (QIAGEN, Hilden, Germany) and then proceeded to quantify the DNA concentration by using the PicoGreen QuantiFluor^®^ dsDNA System (Promega, Madison, USA) on a TECAN Infinite F200 PRO^®^ plate reader (Tecan, Männedorf, Switzerland). We normalized the library to include 20 ng DNA from each sample. Finally, we diluted the library to 3 nM for sequencing. The library was spiked with 5% PhiX sequencing control V3 (Illumina, San Diego, USA). Paired‐end sequencing of the amplicons was performed as recommended by Illumina (MiSeq Reagent Kit v2—Reagent Preparation Guide) and loaded at a final library concentration of 6 pM. Paired‐end sequencing was performed over 2 × 250 cycles.

### 2.5 Bioinformatics analysis

We conducted the demultiplexing and denoising of the samples in the QIIME2 (version 2019.10) pipeline (Bolyen et al., 2019) and used the DADA2 method (Callahan et al., 2016) to get rid of artefacts and chimeras. We trimmed the reads at 200 bp using a mean quality score of 37 in both directions. Only amplicon sequence variants (ASVs) that survived the filtering step were kept for subsequent analyses. We trained a new SILVA V4 Classifier (SSU release 132 515‐806) by using QIIME2 tutorials as a reference (Quast et al., 2012). ASVs were then assigned a taxonomy using the “qiime feature‐classifier classify‐sklearn” function) with the highest resolution possible (level 7). Following the taxonomic assignment, we split the analysis into two parts: we kept the original output of the taxonomy assignment (unfiltered) and then we filtered the chloroplast and mitochondria assigned reads (filtered). This step was necessary to evaluate the effect of the clamps on the percentage of reads that were allocated to the chloroplasts and mitochondria before and after application of the clamps. The script for our analysis is deposited in GitHub (https://github.com/luisvqz/V4_pna_clamps_4_wildlife). Further analyses were performed in R [version 3.4.4 (2018)] by using the *phyloseq* package (McMurdie & Holmes, 2013) in a Linux environment. Plots were generated in R (R Core Team, 2018) by using the package *ggplot2* (Wickham, 2016).

## 3 RESULTS

In bats, we obtained on average 40,278 (±3,719, *n* = 8) reads per individual and in birds 71,367 (±26,981, *n* = 10; Table 1). From early on, we were able to detect that a large percentage of the reads in the unclamped samples were allocated to a few ASVs and, after performing the taxonomic assignment, we were certain that those reads matched known sequences of chloroplasts and mitochondria from publicly available databases. The chloroplast sequences obtained from the bat fecal samples matched 100% with a chloroplast sequence published from the Saguaro Columnar cacti (GenBank Accession Number: KT164771).

**Table 1:** Summary of read counts for each sample before and after the filtering of reads assigned to chloroplasts and mitochondria. The last column summarize the number of reads retained for the downstream microbiome analyses after the filtering.

Sample ID	Species	Clamp use	Total read count	Number of reads assigned to chloroplasts and mitochondria	Filtered read count
140	*L. yerbabuenae*	No	33,128	21,347	11,781
140	*L. yerbabuenae*	Yes	44,350	319	44,031
141	*L. yerbabuenae*	No	31,707	28,320	3,387
141	*L. yerbabuenae*	Yes	36,719	1,986	34,733
142	*L. yerbabuenae*	No	37,856	13,891	23,965
142	*L. yerbabuenae*	Yes	31,372	44	31,328
143	*L. yerbabuenae*	No	32,777	31,243	1,534
143	*L. yerbabuenae*	Yes	31,063	1,072	29,991
144	*L. yerbabuenae*	No	31,564	29,488	2,076
144	*L. yerbabuenae*	Yes	30,579	3,102	27,477
145	*L. yerbabuenae*	No	38,329	22,645	15,684
145	*L. yerbabuenae*	Yes	37,232	173	37,059
181	*L. yerbabuenae*	No	33,516	21,996	11,520
181	*L. yerbabuenae*	Yes	32,589	70	32,519
195	*L. yerbabuenae*	No	30,738	30,295	443
195	*L. yerbabuenae*	Yes	30,421	4,533	25,888
143114	*M. parvulus*	No	76,954	31,062	45,892
143114	*M. parvulus*	Yes	56,890	511	56,379
143120	*M. parvulus*	No	92,269	39,606	52,663
143120	*M. parvulus*	Yes	50,596	196	50,400
143124	*M. parvulus*	No	94,728	36,449	58,279
143124	*M. parvulus*	Yes	39,963	413	39,550
143170	*M. parvulus*	No	121,709	38,511	83,198
143170	*M. parvulus*	Yes	51,766	113	51,653
143185	*M. parvulus*	No	72,513	65,422	7,091
143185	*M. parvulus*	Yes	35,253	7,201	28,052
143193	*M. parvulus*	No	67,647	60,026	7,621
143193	*M. parvulus*	Yes	37,639	10,665	26,974
143195	*M. parvulus*	No	53,607	53,396	211
143195	*M. parvulus*	Yes	34,516	5,108	29,408
143199	*M. parvulus*	No	99,402	84,424	14,978
143199	*M. parvulus*	Yes	41,528	1,585	39,943
143356	*M. parvulus*	No	55,739	52,807	2,932
143356	*M. parvulus*	Yes	31,529	6,966	24,563
143358	*M. parvulus*	No	43,320	42,879	441
143358	*M. parvulus*	Yes	39,658	5,114	34,544

**Figure 3:**
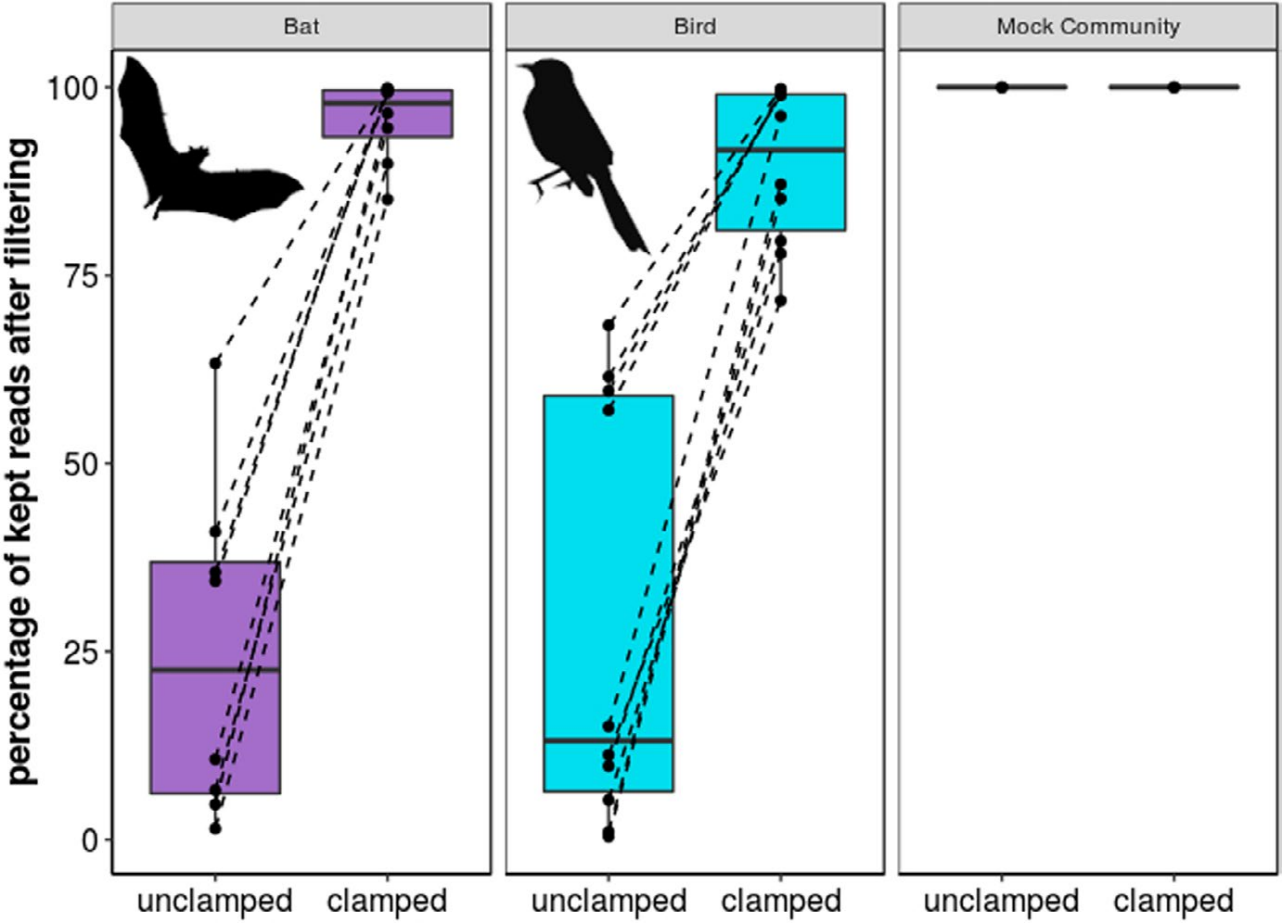
Percentage of reads kept after the filtering out the chloroplast and mitochondrial DNA using QIIME2. Bat samples were clamped by implementing cpPNA/mPNA and birds by using pPNA/mPNA. The Zymo Mock community was treated with cpPNA/mPNA

**Figure 4:**
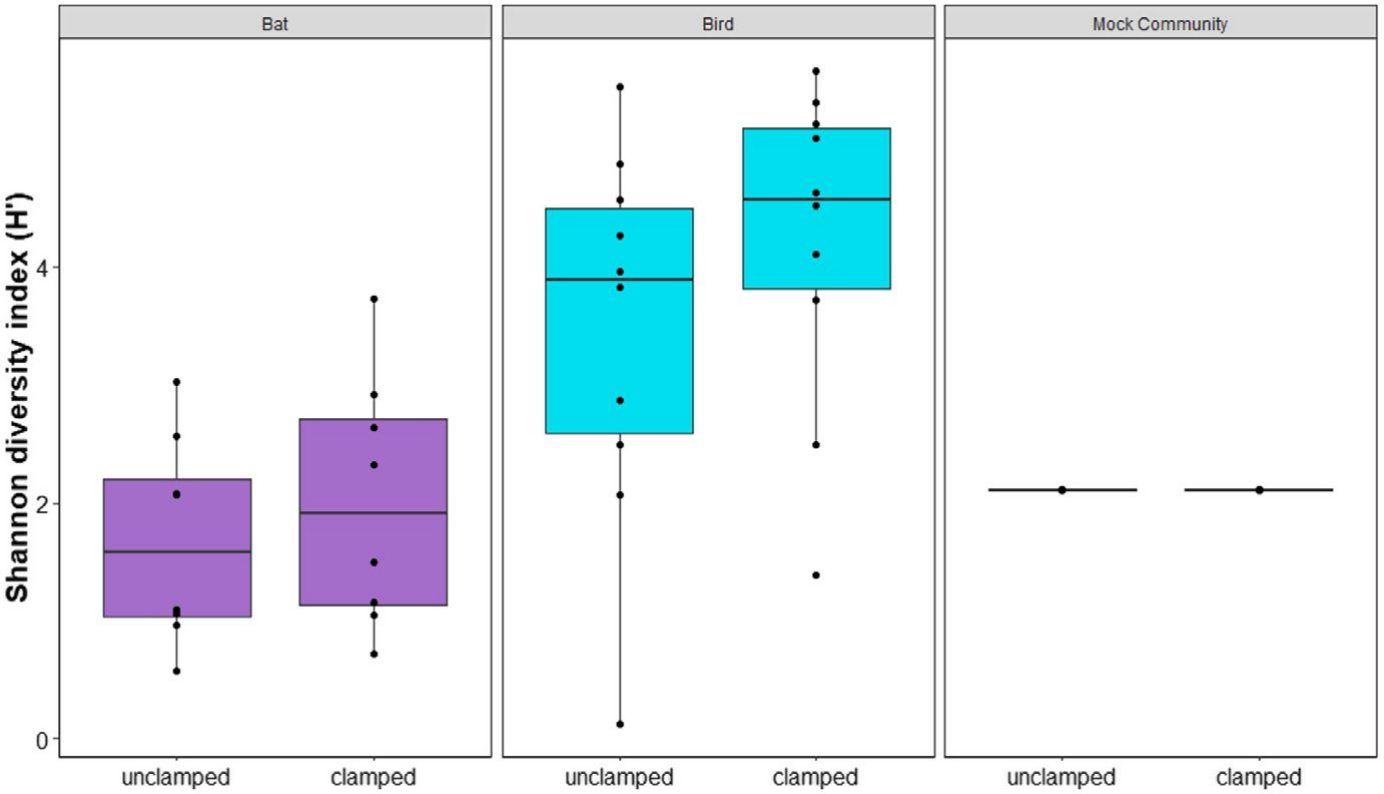
Alpha diversity (Shannon Index) changes in the microbiome community before and after application of clamps in bat, bird, and Zymo Mock community. Bat samples were clamped by implementing cpPNA/mPNA and birds by using pPNA/mPNA. The Zymo Mock community was treated with cpPNA/mPNA

After filtering out the chloroplast and mitochondria assigned reads from the data set, we found that, by using the PNA‐DNA clamps, we were able to retain a significantly larger portion of the reads after the filtering step (Figure 3a). Although the effectivity varied between individual samples, we always detected an improvement of read coverage available for downstream analyses while using the clamps compared with the unclamped results in pairwise comparisons. On average, the percentage of reads kept improved by 13‐fold for the bat (with the clamps cpPNA and mPNA) and by 34‐fold for the bird (cPNA and mPNA) (Table 1). The two extreme cases were the bat sample Lepto‐195 with a 65‐fold improvement and the bird sample MM‐143195 with a 216‐fold improvement. The control samples, that is, the bacterial mock community without chloroplasts and mitochondria, showed no fold change indicating that the use of the clamps did not affect the Zymo Mock community in any way (Figure 3). We also tested for differences in alpha diversity in clamped and unclamped samples and controls. We detected no effect of the clamps on the overall alpha diversity (*p* = .192; Figure 4). Thus, the use of the clamps increased the percentage of reads kept after the subsequent filtering step but did not affect the alpha diversity of the samples.

## 4 DISCUSSION

Challenges associated with plastid contamination represent a major concern in microbiome analyses (Beckers et al., 2016; Gaona et al., 2020; Jackrel et al., 2017). Our results indicate that the use of DNA‐PNA clamps significantly improves the microbiome sequencing output of fecal samples obtained from species with a diet harboring a large amount of chloroplast and mitochondrial DNA. This effect has also been shown by Fitzpatrick et al. (2018) in plant surface microbiomes; however, our study is the first to test the usefulness of clamps in wildlife microbiome studies relying on fecal pellets. Microbiome studies have recently been growing at an accelerated pace. As we move away from model organisms, the diets of the animals under study become more and more diverse. As a rough estimate, 26% of bats and 33% of birds (Ko et al., 2014) follow a plant‐based diet. Therefore, techniques that allow us to bypass the remnant plant material in fecal samples are becoming more and more important for microbiome studies.

One important factor to keep in mind when using PNA‐DNA clamps is the need to have some information about the diet of the study species. PNA‐DNA clamp specificity varies between groups. In our case, we had previous knowledge that, in our study area, the diet of tequila bats consists of almost 100% columnar cacti, particularly from one species, namely *Carnigea gigantea* (LV and MT, personal observation and unpublished data). In the bat case, visual inspection of the fecal pellets also revealed that a large percentage of the pellets was undigested pollen grain clusters. This facilitated the development of the cpPNA clamp thanks to the information available from other studies (Fitzpatrick et al., 2018).

Our technique allows the more cost‐effective use of sequencing capacity. By employing PNA‐DNA clamps, we have been able to target the “true microbiome” more directly and waste fewer reads related to by‐products from the diet of the animal. Having higher read numbers enables better statistical power in the analysis and decreases data losses in the subsequent steps in downstream processing. Other authors have suggested to circumvent this problem by targeting a different region of the 16S rRNA (Copeland et al., 2015). However, previous attempts in our study revealed that sequencing another location did not solve the problem since the contamination was still highly present and abundant after sequencing. Even though the sequencing price tag is becoming cheaper every day (Wetterstrand, 2011), without the PNA‐DNA clamps, we would have had to double or triple or even increase by 10‐fold our sequencing depth to make the latter reasonable enough to allow downstream analyses. The cost of the clamps varies between providers but, in general, the use of the clamps will always be more cost‐effective than aiming at larger sequencing depth. With the expansion of microbiome studies to nonmodel organisms, we believe that additional tools like the one presented in this paper will streamline the future advancement of the field.

## CONFLICT OF INTERESTS

The authors declare no competing interests.

## AUTHOR CONTRIBUTION

**Luis Viquez‐R:** Conceptualization (equal); Formal analysis (lead); Funding acquisition (equal); Investigation (equal); Methodology (equal); Project administration (equal); Writing‐original draft (lead). **Ramona Fleischer:** Data curation (equal); Formal analysis (equal); Investigation (equal). **Kerstin Wilhelm:** Conceptualization (equal); Investigation (equal); Methodology (equal); Writing‐review & editing (equal). **Marco Tschapka:** Conceptualization (equal); Funding acquisition (equal); Supervision (equal); Writing‐review & editing (equal). **Simone Sommer:** Conceptualization (equal); Formal analysis (equal); Funding acquisition (equal); Project administration (equal); Supervision (equal); Writing‐original draft (equal); Writing‐review & editing (equal).

## Data Availability

DNA sequences: Raw data and the necessary inputs for the QIIME pipeline are deposited at Dryad (https://doi.org/10.5061/dryad.4tmpg4f7j). Mapping files, data sheets, and the scripts for all the microbiome analysis (Qiime2 and R) are stored in GitHub (https://github.com/luisvqz/V4_pna_clamps_4_wildlife).
